# Advances in Biomarkers for Diagnosis and Treatment of ARDS

**DOI:** 10.3390/diagnostics13213296

**Published:** 2023-10-24

**Authors:** Ruiqi Ge, Fengyun Wang, Zhiyong Peng

**Affiliations:** 1 Department of Critical Care Medicine, Zhongnan Hospital, Wuhan University, Wuhan 430071, China; 2017302180007@whu.edu.cn; 2 Clinical Research Center of Hubei Critical Care Medicine, Wuhan 430071, China

**Keywords:** acute respiratory distress syndrome, acute lung injury, early diagnosis, biomarker, machine learning

## Abstract

Acute respiratory distress syndrome (ARDS) is a common and fatal disease, characterized by lung inflammation, edema, poor oxygenation, and the need for mechanical ventilation, or even extracorporeal membrane oxygenation if the patient is unresponsive to routine treatment. In this review, we aim to explore advances in biomarkers for the diagnosis and treatment of ARDS. In viewing the distinct characteristics of each biomarker, we classified the biomarkers into the following six categories: inflammatory, alveolar epithelial injury, endothelial injury, coagulation/fibrinolysis, extracellular matrix turnover, and oxidative stress biomarkers. In addition, we discussed the potential role of machine learning in identifying and utilizing these biomarkers and reviewed its clinical application. Despite the tremendous progress in biomarker research, there remain nonnegligible gaps between biomarker discovery and clinical utility. The challenges and future directions in ARDS research concern investigators as well as clinicians, underscoring the essentiality of continued investigation to improve diagnosis and treatment.

## 1. Introduction

Acute respiratory distress syndrome (ARDS) is a critical manifestation of acute lung injury (ALI) characterized by hypoxemic respiratory failure, pulmonary infiltrates on both sides of the chest, and non-cardiogenic pulmonary edema, causing a decline in lung compliance and an inability to exchange gases [1]. ARDS impacts millions of people worldwide and carries a high death rate, along with long-term effects and a complex management approach [2]. Although there have been improvements in supportive measures, such as lung-protective ventilation and fluid management strategies, there is still a lack of targeted treatments to improve clinical outcomes [3]. The heterogeneous etiologies of ARDS have prompted the recognition of multiple subphenotypes, which could lead to personalized treatment for patients [4].

**Figure 1:**
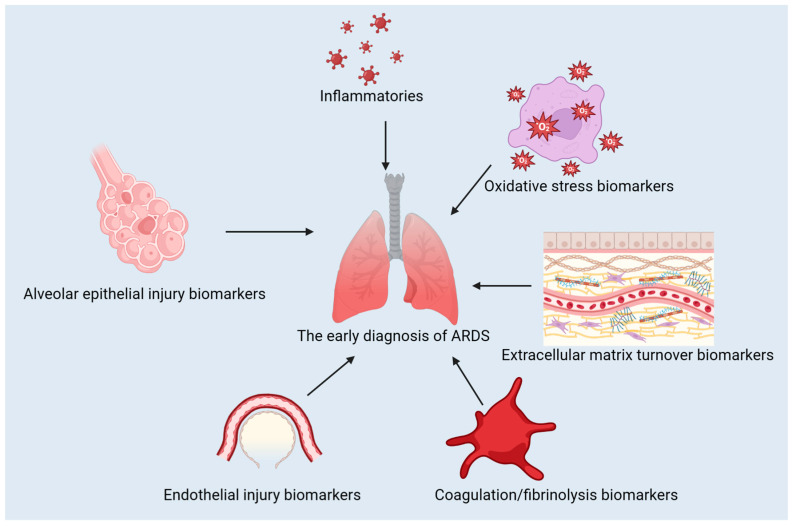
The classification of the biomarkers for ARDS.

The use of biomarkers is pivotal in diagnosing, predicting the course, and treating ARDS. They can be used to distinguish between different types of conditions, evaluate their severity, and track the effectiveness of treatment [5,6]. In this review, we discuss the classification of ARDS biomarkers, advances in their use for diagnosis and treatment, the contribution of machine learning to ARDS biomarker identification, the difficulties in translating biomarker discoveries to clinical practice, and potential future directions for research and development. The classification of the biomarkers discussed in this review is presented in Figure 1.

## 2. Classification of the Biomarkers Related to ARDS

### 2.1. Inflammatory Biomarkers

#### 2.1.1. Interleukin-6 (IL-6) 

Due to its pro-inflammatory and immune-injuring properties, IL-6 is a promising biomarker for ARDS diagnosis and treatment. In a clinical study, of 88 adult patients with ARDS on invasive mechanical ventilation enrolled, the serum levels of IL-6 and granulocyte-colony-stimulating factor in BALF were higher among patients with hyperinflammatory subphenotypes than those with the hypoinflammatory subphenotypes [7]. Moreover, the levels of IL-6 have been found to correlate with the severity of COVID-19, which is known to cause ARDS [8,9,10]. By impeding the cytotoxicity of immune cells in serious COVID-19 cases, IL-6 adds to the seriousness of the illness and the related issues [11].

IL-6 not only serves as a reliable biomarker, but it has also been regarded as a potential target for therapeutics for ARDS. Various studies have studied the possibility of utilizing IL-6 inhibitors to treat COVID-19, as they can lessen cytokine release syndrome (CRS) and reduce the load of the virus [12,13]. As an illustration, the application of tocilizumab, an IL-6 inhibitor, has been demonstrated to better the outcomes of COVID-19 patients [9]. Apart from directly blocking IL-6, an RCT involving a total of 43 patients with ARDS proved that mesenchymal stromal cells can lower the amount of IL-6 [14]. In a randomized clinical trial involving 36 people with moderate to severe ARDS caused by COVID-19, alpha-1 antitrypsin was found to decrease IL-6 in serum [15]. Moreover, IL-6 has been proposed as a potential target for modifying the immune response in ARDS patients. The IL-6-p-STAT3-p63-JAG2 pathway has been validated as a promising therapeutic target in ARDS [16]. 

#### 2.1.2. Interleukin-8 (IL-8)

IL-8, a pro-inflammatory biomarker, has a substantial influence on the pathophysiology of ARDS attributing to its participation in the mobilization and activation of neutrophils. Elevated IL-8 concentrations have been noted in ARDS patients, indicating its potential as a biomarker for the early diagnosis and observation of the illness’s progression [17]. A link between IL-8 and the emergence of ARDS in those with severe traumatic brain injury has been established [17]. A case series of ARDS due to COVID-19 revealed that higher levels of IL-8 in the blood were associated with the hyperinflammatory phenotype of ARDS [18].

Numerous investigations into COVID-19 have highlighted IL-8 as an indicator of the severity and mortality of the disease. An increase in IL-8 in the serum of COVID-19 patients has been linked to an increased risk of death [19,20]. Additionally, elevated concentrations of IL-8 in the blood have been associated with prolonged sickness in people suffering from severe COVID-19 [21]. Research has indicated that IL-8 plays a role in causing prothrombotic neutrophil phenotype in serious COVID-19, resulting in blood clotting and an increase in respiratory difficulty [22]. In a case series of severe COVID-19 patients who required mechanical ventilation, intravenous infusions of mesenchymal stem cells were reported to lower the serum IL-8 and mitigate respiratory distress [23].

#### 2.1.3. Tumor Necrosis Factor-Alpha (TNF-α)

TNF-α, a pro-inflammatory cytokine, is of the utmost importance in the development of ARDS, responsible for lung inflammation, alveolar epithelial injury, and endothelial cell damage [24,25]. Evidence suggests that TNF-α plays a part in inflammatory cell death, tissue destruction, and fatality in cases of COVID-19 infection and cytokine shock syndromes [26]. Additionally, research has suggested that TNF-α in combination with IL-17A could be a potential biomarker for ARDS and mortality in obese COVID-19 patients [27]. 

Utilizing intravital microscopy in a model of mouse sepsis, the research ascertained that the speed of degradation in the pulmonary microvascular glycocalyx due to endotoxemia was TNF-α-dependent [28]. In the Intestinal Ischemia-Reperfusion (IIR)-caused ALI model, the JNK/FoxO3a pathway was set off by TNF-α, resulting in a deferment of polymorphonuclear neutrophils apoptosis and, as a result, promoting the ALI developed by IIR [29].

High serum levels of soluble TNF-α receptors have been correlated with mortality in the ICU for COVID-19 patients [30]. On the other hand, diminishing it might result in a better outcome. In a study of endotoxin-induced ARDS, Nimbolide, a bioactive compound, was demonstrated to protect against ARDS by restraining TNF-α-activated NF-κB and HDAC-3 nuclear translocation [31]. It has been stated that the amalgamation of TNF-α and IFN-β stimulates human airway epithelial cell death through apoptosis and pyroptosis, which ultimately results in lung injury in ARDS [25].

#### 2.1.4. The Neutrophil Response Index (NEUT-RI)

The Neutrophil Response Index (NEUT-RI), a measure of neutrophil activation, is now accessible in many laboratories and used to assess the systemic inflammatory response associated with inflammatory and autoimmune diseases including pemphigus and autoimmune hepatitis [32,33]. Likewise, it is associated with the gravity of COVID-19, including the requirement for mechanical ventilation and the risk of death [34]. Also, NEUT-RI has been utilized to characterize and differentiate convalescent patients from those with active COVID-19, illustrating its potential to monitor disease progression and recovery [35].

### 2.2. Alveolar Epithelial Injury Biomarkers

#### 2.2.1. Receptor for Advanced Glycation End Products (RAGE) 

RAGE, a receptor for advanced glycation end-products, has been discovered to be a crucial biomarker for the diagnosis and treatment of ARDS. Progress in grasping the part played by RAGE in ARDS’s pathogenesis has brought new possibilities for hopeful therapeutic interventions.

The activation of the multi-ligand RAGE cell-surface receptor is associated with inflammatory processes and oxidative stress [36]. There is evidence to advocate that heightened RAGE activation is related to multiple comorbidities, such as pulmonary diseases, and it is deemed as a risk factor for severe COVID-19 [37]. Through the NLRP3 inflammasome/TXNIP axis, RAGE activation in ARDS encourages the activation of alveolar macrophages, culminating in ALI [38].

Research has indicated that sRAGE may be utilized as a biomarker to gauge the severity of COVID-19, from the necessity of mechanical ventilation, ARDS, to mortality [39]. Higher plasma sRAGE levels are correlated with a higher mortality rate in ARDS sufferers [40]. This discovery underlines the possibility of sRAGE being a predictive biomarker in ARDS. The utilization of RAGE for therapeutic intervention has become increasingly popular recently, and numerous small-molecule inhibitors have been developed to target RAGE and its associated downstream pathways [41]. The inhibition of RAGE via Ager gene knockout in mice or siRAGE silencing in type II alveolar epithelial cells (ATII) has attested that RAGE plays a key role in the pathogenesis of type II alveolar epithelial cell injury and that the inhibition of RAGE could abate LPS-induced lung injury [42]. Beyond that, a peptide that is derived from HMGB1 (high mobility group box 1) and is a recombinant RAGE antagonist has been observed to have anti-inflammatory properties and reduce ALI in preclinical studies [25]. These innovations offer propitious approaches for the development of novel ARDS therapies aimed at RAGE and its related pathways.

#### 2.2.2. Surfactant Protein-D (SP-D) 

It is known that SP-D, an integral element of pulmonary surfactant, is essential for the innate immune response in the lungs. More recently, SP-D has been revealed as a biomarker of alveolar epithelial injury in those suffering from ARDS [43]. Agustama et al.’s investigation demonstrated a clear connection between SP-D serum levels and ARDS severity and mortality in COVID-19 patients, suggesting the potential of SP-D as a diagnostic and prognostic tool for ARDS management [44]. In COVID-19 patients who had ARDS and were treated with mechanical ventilation, higher serum SP-D levels were associated with reduced respiratory compliance and higher ARDS severity, and may serve as a diagnostical biomarker [45].

Apart from its diagnostic capabilities, studies have been conducted to explore the use of SP-D in drug delivery systems for lung diseases in premature infants. Attias Cohen et al. examined the use of SP-D-loaded poly(lactic-co-glycolic acid) (PLGA) nanoparticles for drug delivery, and unraveled the potential of SP-D as a therapeutic agent [46]. García-Mouton et al. advocated the use of an interface-aided transporter to introduce SP-D into the airways, potentially applying it to treat respiratory diseases [47].

Exogenous pulmonary surfactants, such as SP-A and SP-D, have been put forward as a potential adjunctive therapy for COVID-19, further underlining the centrality of SP-D in lung injury and inflammation [48]. Other research has looked into the function of SP-D as an indicator of COVID-19 [49] and its predicting ability throughout the course of the infection [50]. 

#### 2.2.3. Clara Cell Secretory Protein (CCSP or CC16)

CC16, a biomarker of alveolar epithelial injury, has the potential to diagnose and predict the prognosis of ARDS [51]. Almuntashiri et al. studied the prognostic value of CC16 cut-points in a distinct cohort of ALTA (ALI and ARDS patients), illustrating the capability of CC16 as a promising biomarker for ARDS [51].

In a swine model of multi-trauma, CC16 was demonstrated to be a promising lung injury indicator, further validating its part in gauging lung injury and ARDS development [52]. sEV-CC16, encapsulated in extracellular vesicles, has the potential to reduce the inflammatory and DNA damage responses in LPS or bacteria-induced ALI mouse models by attenuating the NF-κB pathway [53].

To ascertain their predictive capacity in COVID-19, the kinetics of CC16 and SP-D pneumoproteins have been explored. It has been found that CC16 may be a reliable indicator of the severity and potential outcomes of COVID-19 [50].

### 2.3. Endothelial Injury Biomarkers

#### 2.3.1. Angiopoietin-2 (Ang-2) 

The renin–angiotensin system (RAS) is a major contributor to the development of ARDS [54]. The utilization of the most current biomarkers concerning the Ang axis of the RAS has aided in the identification and treatment of ARDS. A systemic review and meta-analysis of 18 pertinent studies and 27 datasets revealed that Ang-2 has potential diagnostic and prognostic capabilities regarding ARDS, especially among Chinese people [55]. Multivariable models from a cohort of 757 patients with sepsis showed that Ang-2 was linked to the development of ARDS and 30-day mortality, making it a promising biomarker and an attractive target for vascular injury [56]. It has been highlighted that the ACE2/Ang-(1-7)/MasR axis is a major component in the development of pulmonary fibrosis in ARDS [57]. This axis serves to counterbalance the pro-inflammatory and pro-fibrotic effects of the ACE/Ang II/AT1R axis, thereby providing a shield against inflammation and fibrosis in lung tissues. It has been proven that the activation of the ACE2/Ang-(1-7)/MasR axis has protective effects on organs, including protection against endothelial dysfunction in the lungs [58,59].

Experiments conducted on Mycoplasma pneumoniae-infected mice unveiled the capacity of Angiotensin-(1-7) peptide hormone in mitigating inflammation and the amount of pathogens, adding its weight as a potential therapy for ARDS [60]. An association between elevated Ang-2 levels and a greater mortality rate has been observed in ARDS patients [61]. The results of a meta-analysis of 10 prospective cohort studies suggested that higher Ang-2 concentrations are linked to a higher mortality risk, thus emphasizing the potential of Ang-2 as a biomarker for ARDS prognosis [61]. And, it has been observed that increased Ang-2 levels are associated with a more pressing need for mechanical ventilation following cardiac surgery, suggesting its involvement in postoperative pulmonary complications [62].

#### 2.3.2. Von Willebrand Factor (vWF) 

Endothelial injury in ARDS can be determined through von Willebrand factor (vWF), a notable biomarker. vWF, a glycoprotein, is of paramount importance in the clotting of blood and adhesion of platelets; its malfunction can cause various endothelial diseases. High levels of vWF are related to the severity of respiratory diseases such as COVID-19, which attests to its potential as a marker of endothelial cell activation and inflammation [63]. A retrospective analysis showed that plex reduced the excess of vWF and increased the activity of ADMTS 13, leading to the adjustment of the ratio of ADMTS 13 / vWF and thus reducing the risk of immunothrombosis in COVID-19 patients [64].

Investigations have revealed correlations between vWF levels and other signs of inflammation, as well as the great quantity of extracellular DNA in arterial thrombi [65]. Endotheliopathy, which is defined by high vWF antigen concentrations, has been linked to poor prognoses in COVID-19 patients, thus confirming the medical importance of vWF in ARDS and similar medical conditions [66]. It is important to bear in mind, however, that most of the studies conducted on vWF as a biomarker for ARDS have focused on COVID-19, and additional research is needed to assess its significance in other etiologies of ARDS.

#### 2.3.3. Intercellular Adhesion Molecule-1 (ICAM-1)

In ARDS, ICAM-1 has been identified as a vital biomarker of endothelial injury. It is a glycoprotein on the cell surface that enables the adhesion and transmigration of leukocytes across the vascular endothelium and is integral to inflammatory processes [67].

An association between ICAM-1 expression and lung inflammation as well as ARDS progression has been observed in ARDS. Among pediatric patients with ARDS, an observational study found that soluble ICAM1 had the most powerful positive association with the worsening of lung injury throughout the entire study period [68]. In an ALI mouse model, the depletion of BAP31 caused a marked decline in neutrophil attachment to endothelium cells, which was largely ascribed to the MyD88/NF-κB-dependent decrease in ICAM-1 [69]. A mouse study investigating the effects of MMI-0100, a peptide inhibitor, showed that it was able to reduce endothelial ICAM-1 expression and thus reduce lung inflammation [70]. Therefore, targeting ICAM-1 expression may prove to be a viable therapeutic option for ARDS.

For infants suffering from neonatal respiratory distress syndrome, a blend of pulmonary surfactant and high-frequency oscillatory ventilation has been found to have an effect on CD cells and ICAM-1 level, thereby impacting their immune system [71]. MALAT1, a type of long non-coding RNA, has been linked to the worsening of ARDS via an increase in ICAM-1 expression as a result of the reduced activity of microRNA-150-5p, implying a possible molecular mechanism for ICAM-1’s role in the development of ARDS [72], which is consistent with the findings of a clinical study on COVID-19, where the elevation of ICAM-1 level was observed in non-survivors [73].

### 2.4. Coagulation/Fibrinolysis Biomarkers

#### 2.4.1. Plasminogen Activator Inhibitor-1 (PAI-1) 

Plasminogen activator inhibitor-1 (PAI-1) is an essential biomarker in the fibrinolysis/coagulation pathway linked to ARDS, being a serine protease inhibitor whose role is to control fibrinolysis by inhibiting tissue plasminogen activator (tPA) and urokinase plasminogen activator (uPA) [74]. Elevated PAI-1 levels can consequently impair fibrinolysis and be a factor in the occurrence of ARDS. Investigating the single nucleotide polymorphisms (SNPs) of PAI-1 and its serum levels in 181 persons with sepsis and ARDS revealed that genetic polymorphisms of PAI-1 could alter the serum levels of PAI-1, potentially resulting in death by affecting neutrophil activity [75].

Recent findings proved that SARS-CoV-2 spike protein can promote the production of endothelial PAI-1, suggesting its potential role in ARDS induced by COVID-19 [76]. In alveolar epithelial cells stimulated by LPS, the suppression of NF-κB reduced the expression of PAI-1, while increasing the output of activated protein C, signifying the role of PAI-1 in ARDS development [77].

#### 2.4.2. Thrombomodulin

Thrombomodulin is an indispensable biomarker in the coagulation/fibrinolysis pathway associated with ARDS. This glycoprotein found on endothelial cell membranes has a critical role in anticoagulation, as it binds to thrombin and then activates protein C [78]. Evidence from a study on rats with septic peritonitis suggests that recombinant human thrombomodulin (rhTM) can reduce mortality and lessen the severity of ALI, likely due to its anti-inflammatory and anticoagulant properties [79]. A systematic review and meta-analysis, involving a total of 13 studies, was conducted to assess the association and predictive value of soluble thrombomodulin (sTM) levels for mortality in ARDS patients; the results showed that elevated sTM levels were linked to a higher mortality risk [80]. The results of another study that included children with acute respiratory failure who were receiving mechanical ventilation showed that higher thrombomodulin levels were linked to higher mortality and organ dysfunction [81]. Promising results have been observed regarding the use of recombinant human thrombomodulin for pediatric cases of severe ARDS associated with DIC caused by pneumonia [82]. 

#### 2.4.3. D-Dimer

Research has been conducted in ARDS on D-dimer, a biological marker that originates from the breakdown of fibrin. The level of D-dimer is regularly utilized as a measure of activated coagulation and fibrinolysis. Higher D-dimer amounts have been linked to the severity and outcome of COVID-19 cases [83,84,85,86]. A systematic review has compared D-dimer concentrations in ARDS patients not suffering from COVID-19 and those suffering from COVID-19, demonstrating that D-dimer concentrations are significantly elevated in the latter [87]. A study has uncovered that platelet distribution width and augmented D-dimer at admission can anticipate the subsequent development of ARDS in COVID-19 patients [88]. 

### 2.5. Extracellular Matrix Turnover Biomarkers

#### 2.5.1. Matrix Metalloproteinase-9 (MMP-9) 

Matrix metalloproteinases (MMPs) are a family of endopeptidases that are reliant on zinc for them to work and are in charge of the decomposition of extracellular matrix (ECM) components. MMP-9, often referred to as gelatinase B, is deeply involved in restructuring ECM, tissue repair, and inflammatory proceedings [89]. Increased concentrations of MMP-9 have been linked to a variety of respiratory diseases, such as COVID-19. MMP-9 levels in the serum of those with COVID-19 have been observed to be elevated, with this associated with the seriousness of the sickness and the probability of death [90,91]. According to Antoine Rebendenne et al.’s research, individuals with a high viral load, weak antibody response, and high levels of MMP-9 have prolonged pulmonary pathology due to COVID-19 [92]. The involvement of MMP-9 in the etiology of severe COVID-19 lung illness was determined through its interaction with MMP-8, the modification of the immune response through HLA-G exfoliation, and oxidative stress [93].

#### 2.5.2. Tissue Inhibitor of Metalloproteinase-1 (TIMP-1)

Tissue inhibitor of metalloproteinase-1 (TIMP-1) is a biomarker of relevance to extracellular matrix turnover, and plays a key role in the control of matrix metalloproteinases (MMPs), which are responsible for the breakdown of extracellular matrix components. Maintaining a proper equilibrium between MMPs and TIMPs is fundamental for tissue homeostasis, and the disruption of this balance has been associated with specific pathological processes, including ALI and ARDS [94].

Almuntashiri et al.’s study showed that high levels of plasma TIMP-1 are linked to ALI in female patients, whereas no such association was observed in male patients. This emphasizes the need to take gender differences into account when examining biomarkers for ARDS and other lung injuries [94].

Apart from its use as a diagnostic biomarker, TIMP-1 has been targeted for therapeutic applications in ARDS. Chernikov et al. studied the efficiency of siRNA-mediated TIMP-1 silencing as an approach to reduce the inflammatory phenotype during ALI. The authors demonstrated that by dampening TIMP-1, inflammation was lessened and respiratory function was improved in an ALI experiment, pointing to the possibility that TIMP-1 manipulation could be a potential remedy for ARDS [95].

### 2.6. Oxidative Stress Biomarkers

#### Malondialdehyde (MDA)

The formation of MDA occurs when polyunsaturated fatty acids are assaulted by free radicals during the lipid peroxidation process, leading to cell membrane damage and impaired cellular function. In ARDS, an increase in MDA is an indication of greater oxidative stress and lipid peroxidation, and these are the cause of the alveolar damage and inflammatory response associated with the condition. A cohort study of patients with sepsis and ARDS indicated that circulating MDA was higher in patients with ARDS compared with those without any organ failures [96]. Monitoring MDA levels can offer valuable references to the extent of oxidative damage and the efficacy of antioxidant therapies in ARDS [97].

Targeting oxidative stress and lipid peroxidation represents a promising therapeutic strategy in ARDS. Compounds such as Ferrostatin-1, which inhibits ferroptosis, have exhibited potential in alleviating LPS-induced ALI by reducing MDA levels and mitigating oxidative damage [98]. Obacunone, a natural compound, has been established to alleviate LPS-induced ALI by decreasing ROS and MDA production and activating Nrf2-dependent antioxidant responses [99].

### 2.7. Machine Learning and ARDS Biomarkers

**Figure 2:**
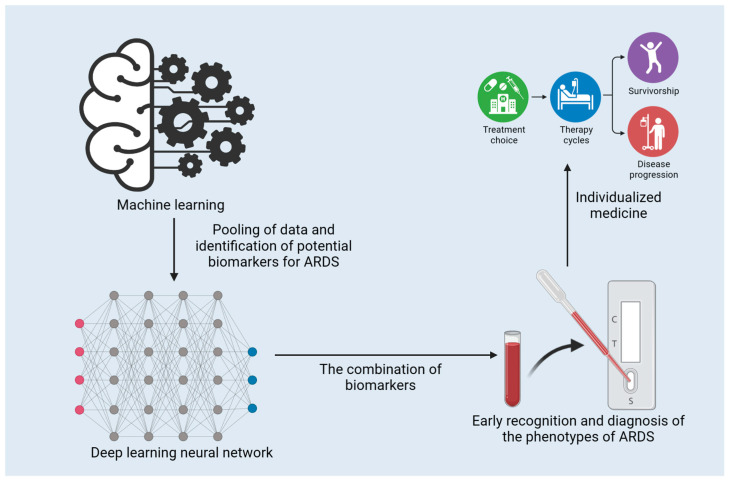
The application and function of machine learning in the early diagnosis of ARDS.

Machine learning (ML) has presented evidence of its capacity to improve our understanding and management of ARDS. ML techniques have been implemented to predict the occurrence of ARDS, classify clinical phenotypes, and investigate the associations between biomarkers and ARDS outcomes [100,101,102,103]. The application and function of ML in the early diagnosis of ARDS are presented in Figure 2. ML algorithms have been created to predict ARDS from electronic health record (EHR) data, and a study by Lam et al. has demonstrated encouraging results with recurrent neural networks [101]. Afshar et al. went on to devise a computable phenotype for ARDS using natural language processing and ML, which displayed increased sensitivity and specificity in comparison to traditional techniques [104]. The clinical management of COVID-19 involves Gattino’s categorization of patients into Type L and Type H [105]. ML can be employed to expedite this categorization process. Bai et al. adopted ML to predict sepsis-associated ARDS in the ICU and identify clinical phenotypes with varied treatment results [106].

The application of ML models has enabled the detection of ARDS subphenotypes, thus providing more individualized management for patients. Sinha et al. developed ML classifier models to pinpoint ARDS phenotypes based on available clinical data, which showed considerable distinctions in clinical characteristics, biomarker levels, and outcomes [103]. Maddali et al. tested the usefulness of these subphenotypes in various cohorts, revealing that they had connections to diverse responses to positive end-expiratory pressure (PEEP) and fluid management plans [102].

Utilizing machine learning, Calabrese et al. conducted an ML-driven investigation into alveolar and vascular harm in COVID-19 respiratory failure, examining the role of biomarkers in ARDS patients in the process [107]. This approach could help identify new biomarkers and therapeutic targets for patients with ARDS. Despite the progress made in the use of an ML biomarker in the treatment of ARDS, there are still challenges, including the translation of the results of an ML into clinical practice and the need to have a larger and more diverse dataset, and to develop robust models and models that are interpretable. Ongoing exploration in this area can establish a pathway and refine the detection and treatment of ARDS [100,108,109].

## 3. Challenges and Future Directions

### 3.1. Gaps between Biomarker Discovery and Clinical Utility

Although many biomarkers connected to ARDS have been uncovered, there is still a notable gap between pinpointing them and their clinical application [110]. The heterogeneity of ARDS, caused by different etiologies and the production of a variety of clinical phenotypes, is a challenge and makes it hard to identify a biomarker or set of biomarkers that can be used as a universal diagnostic, prognostic, or therapeutic tool [111,112]. The study frequency of each biomarker for ARDS discussed in this review is presented in Table 1.

An additional problem is the absence of standardization in biomarker measurements and cut-offs, which can cause discrepancies in outcomes and complexity in deciphering data. Establishing standard approaches for biomarker measurement and evaluation is essential to guarantee their medical relevance [113].

More than that, the majority of detected biomarkers are not specific to ARDS. Some biomarkers, such as IL-6, IL-8, and TNF-α, can be raised in multiple types of inflammation, limiting their ability to differentiate ARDS from other conditions [110]. As a result, more precise biomarkers or a blend of biomarkers that can distinguish ARDS from other inflammatory disorders is a necessity.

Translating the results of preclinical studies to humans is yet another issue. Numerous biomarkers that appear to be successful in preclinical studies do not reproduce the same effects in trials conducted with humans [114]. This gap can be explained by the varying pathophysiology of ARDS between animal models and humans, along with variations in how studies are conducted and the types of patients involved. Finally, it is imperative to establish the validity of biomarkers through extensive and properly structured clinical trials. Unfortunately, such studies often require a substantial amount of funding and resources, making it difficult to translate biomarker research into clinical use [110,115].

### 3.2. Possible Approaches for Further Research and Development

Future investigations and innovations could concentrate on certain principal areas to refine ARDS diagnosis and treatment.

Perfecting biomarker panels to enhance accuracy and sensitivity is paramount. By combining multiple biomarkers associated with different aspects of ARDS, for instance, inflammation, epithelial harm, endothelial injury, coagulation, fibrinolysis, extracellular matrix turnover, and oxidative stress, more accurate and reliable diagnostic and prognostic instruments can be established [113].

Examining the genetic and genomic influences regarding ARDS risks and mortality can enlarge our perception of the fundamental mechanisms of the syndrome and individuals’ susceptibility to it [112,116]. Utilizing genomics and other omics advances can facilitate the identification of distinct targets for intervention and the development of personalized medicine approaches [111].

Incorporating machine learning and AI techniques to investigate extensive datasets, including clinical data, biomarker data, and omics data, can assist in discovering complex designs and associations that may not be immediately noticeable through customary statistical approaches [117]. Utilizing these computational techniques can facilitate the production of more precise diagnostics, prognostics, and treatment plans for ARDS.

Researching new therapeutic targets, including those associated with endothelial dysfunction, vascular injury, and inflammation, can provide innovative intervention strategies and better patient outcomes [114,118,119]. Studying the molecular dynamics of ARDS and their interactions with various biomarkers could potentially unravel new treatments and approaches to alleviate the syndrome [115,120].

## 4. Conclusions

### 4.1. Summary of Advances in ARDS Biomarkers

The progress made in recognizing ARDS biomarkers affords us a wider viewpoint into the disease’s pathophysiology, diagnosis, and therapy. Furthermore, the documentation of these advances is imperative for upcoming studies. To achieve this objective, we looked over the related literature and divided the above-noted biomarkers into six groups: inflammatory, alveolar epithelial damage, endothelial damage, coagulation/fibrinolysis, extracellular matrix transformation, and oxidative stress biomarkers [113]. The utilization of these biomarkers has been essential in recognizing ARDS subphenotypes, ultimately leading to more personalized treatment [4]. By using machine learning and other advanced computational methods, our ability to examine and comprehend these biomarkers has increased, which has resulted in more accurate predictive models for ARDS risk and patient outcomes [6].

### 4.2. Importance of Continued Research for Improving Diagnosis and Treatment of ARDS

Despite huge progress in understanding ARDS biomarkers, there are still obstacles and deficiencies in translating these findings into the clinical arena [3]. To gain a better understanding of ARDS and its varied pathophysiological mechanisms, it is essential to carry out further research to refine and validate these biomarkers for improved diagnosis, prognosis, and individualized treatment strategies. Additionally, looking into novel biomarkers and their integration into predictive models will help us progress. This ongoing research will ultimately result in better patient results by allowing clinicians to identify high-risk patients and customize treatments to suit individual patient needs [1].

## Figures and Tables

**Table 1:** The study frequency of each biomarker for ARDS discussed in this review.

	Routinely Tested	Commonly Tested	Less Frequently Tested	Research-Specific
Inflammatory biomarkers	IL-6, IL-8, TNF-α	ICAM-1		
Alveolar epithelial injury biomarkers			SP-D, CCSP	RAGE
Endothelial injury biomarkers		vWF	Ang-2	
Coagulation/fibrinolysis biomarkers	D-dimer	PAI-1, Thrombomodulin		
Extracellular matrix turnover biomarkers			MMP-9, TIMP-1	
Oxidative stress biomarkers				MDA

## Data Availability

This study did not generate or analyze any new data; therefore, data sharing does not apply.
